# Association between socioeconomic status and risk of chronic obstructive pulmonary disease in China: a prospective cohort study

**DOI:** 10.1186/s12889-024-19490-x

**Published:** 2024-07-31

**Authors:** Yujie Hua, Xikang Fan, Mengshi Yang, Jian Su, Jia Guo, Jianrong Jin, Dianjianyi Sun, Pei Pei, Canqing Yu, Jun Lyu, Ran Tao, Jinyi Zhou, Yan Lu

**Affiliations:** 1 Department of Non-communicable Chronic Disease Control and Prevention, Suzhou Center for Disease Control and Prevention, Suzhou, 215004 China; 2 Jiangsu Provincial Center for Disease Control and Prevention, 172 Jiangsu Road, Nanjing, 210009 Jiangsu China; 3 Department of Epidemiology and Health Statistics, Southeast University, Nanjing, 210009 China; 4 Wuzhong District Disease Control and Prevention Centre, Suzhou, 215000 China; 5 Department of Epidemiology & Biostatistics, School of Public Health, Peking University, Xueyuan Road, Haidian District, Beijing, 100191 China; 6 Peking University Center for Public Health and Epidemic Preparedness and Response, Beijing, 100191 China; 7 Key Laboratory of Epidemiology of Major Diseases (Peking University), Ministry of Education, Beijing, 100191 China

**Keywords:** Socioeconomic status, Chronic obstructive pulmonary disease, COPD, Prospective cohort study, China Kadoorie Biobank

## Abstract

**Objective:**

Socioeconomic status (SES) has been proven to be associated with chronic obstructive pulmonary disease (COPD) in Western populations, but the evidence is very limited in China. This study aimed to investigate the association between SES and the risk of COPD incident.

**Methods:**

This study was based on the China Kadoorie Biobank (CKB) project in Wuzhong District, Suzhou. A total of 45,484 adults aged 30–79 were included in the analysis during 2004–2008. We used Cox proportional hazard models to investigate the association between SES and the risk of COPD. Household income, education, private property and consumption potential was used to measure SES. Incident COPD cases were ascertained using hospitalization records, death certificates, and active follow-up.

**Results:**

A total of 524 COPD cases were identified during a median follow-up of 11.2 years. Household income was inversely associated with the risk of COPD (*P*_trend_<0.005). The adjusted hazard ratios (95% confidence intervals) for incident COPD were 0.88 (0.69–1.14), 0.77 (0.60–0.99), and 0.42 (0.31–0.57) for participants with annual household income of 10,000 ~ 19,999 yuan, 20,000 ~ 34,999 yuan and ≥ 35,000 yuan respectively, in comparison to participants with an annual household income < 10,000 yuan. Furthermore, we found that education level, refrigerator use, private toilet, private phone, and motor vehicle were adversely associated with COPD risk, while ownership of newly renovated flats was positively correlated with COPD incident.

**Conclusions:**

This prospective study suggests that SES is associated with the risk of COPD in Chinese adults. Population-based COPD prevention strategies tailored for people with different SES could help reduce the burden of COPD in Chinese.

## Introduction

Chronic obstructive pulmonary disease (COPD) is a common, preventable and treatable condition characterized by persistent airflow restriction [1]. It is a leading cause of morbidity, mortality, and health care use worldwide, especially in developing countries [2–5]. The disease burden of COPD in China is found greater than in developed countries [6]. The direct medical expenses of COPD range from 72 to 3,565 USD per capita per year, accounting for 33.33–118.09% of the local average annual income [7]. Moreover, with the great changing in aging population, economic transformation and lifestyles over the past few decades, the burden of COPD in China is expected to continue to rise significantly [8–10]. Therefore, exploring risk factors of COPD is particularly important to take public health strategies to prevent and control this disease.

Several recent studies reported that middle or high socioeconomic status (SES) is associated with a lower risk of COPD [11, 12]. SES is a comprehensive indicator of income, education and occupation, which reflects the social status of members of society. SES is proved to be associated with non-communicable disease (NCDs) risk, while the strength and direction of SES-NCD associations differ within and between countries [13–15]. However, there was a lack of high-quality research on COPD and SES in China. The majority of current studies were cross-sectional studies, which suggested that SES has an impact on mortality in patients with COPD. But few empirical studies have evaluated the effects of individual SES on COPD morbidity in community residents [12, 16].

Therefore, based on a prospective cohort study, this study aimed to identify the relation between SES and the incidence of COPD, and evaluate the potential impact of SES disparity on the COPD population among Chinese adults.

## Methods

### Study design and participants

This study was based on the China Kadoorie Biobank (CKB) project in Wuzhong District of Suzhou, Jiangsu Province. The baseline survey was conducted in ten regions of China, including five urban and five rural sites. Wuzhong District is one of the urban sites. 53,269 residents aged 30–79 years without disabilities were recruited during 2004–2008. Questionnaires, physical examination and blood samples were used to collect baseline information of the participants. More details on the study design, survey method, questionnaire and long-term follow-up of this study have been described elsewhere [17, 18]. Measurements of forced expiratory volume in 1 s (FEV_1_) and forced vital capacity (FVC) were made using a handheld Micro Spirometer by trained technicians following recommended procedures [19]. After excluding individuals who had airflow obstruction (*n* = 5,906), which defined as a ratio of FEV_1_/FVC less than the lower limit of normal [20], self-reported doctor-diagnosed chronic bronchitis, emphysema or pulmonary heart disease (*n* = 1,843), missing (*n* = 10) or aberrant value (FEV_1_/FVC > 1, *n* = 26) of FEV_1_/FVC at baseline, 45,484 individuals were finally included in our analysis.

This study was approved by Ethical Review Committee of the Chinese Center for Disease Control and Prevention (Beijing, China) and the Oxford (UK). All participants provided written informed consent before the survey.

### Assessment of socioeconomic status

In this study, SES was measured by eight items: self-reported annual household income, education attainment level, newly renovated flats within 5 years, private toilet, private phone, motor vehicle, holiday during last 5 years and refrigerator use (years). Annual household income was the main SES variable in our analysis and was assessed by questioning participants about their total household income last year (< 10,000 yuan, 10,000–19,999 yuan, 20,000–34,999 yuan, or ≥ 3,5000 yuan). Education attainment level was assessed by questioning his/her highest level of school education ever received and categorized into no formal school or less than primary school, primary or middle school, and high school or above. Refrigerator use was assessed by the question: ‘How many years have you had a refrigerator in your home?’ and categorized into four groups: 0 years, 1 to 9 years,10 to 19 years, or ≥ 20 years. The other variables, including newly renovated flats within 5 years, private toilet, private phone, motor vehicle, and holiday during last 5 years, were defined as dichotomous variables.

### Assessment of covariates

Covariates considered in this analysis were also collected at baseline by trained health workers, including sociodemographic characteristics (age, gender and marital status), lifestyle behaviors (alcohol consumption, smoking status, dietary information and physical activity), personal health and medical history (body mass index and prevalent respiratory disease at baseline), and indoor air pollution (passive smoking, cooking fuel and heating fuel).

Standing height and weight were measured by qualified personnel with calibrated tools. Body mass index (BMI) was calculated as body (weight in kilograms)/ (height (in meters)^2^). Smoking status was categorized as never (no more than 100 cigarettes in a lifetime), occasional, ex-regular smoker (stopped smoking for at least 6 months), and current (currently smoking most days or every day). Alcohol use was categorized as non-drinker (never or almost never), former drinker (currently not drinking at all, but previously had a history of weekly alcohol use for at least one year), occasional drinker (currently drinking on special occasions, during special seasons, or monthly, but less than once a week), and regular drinker (currently drinking at least once a week). Prevalent respiratory disease included pulmonary tuberculosis and asthma. The level of total physical activity was calculated as equivalent task hours per day (MET-h/d) spent on occupational, commuting, domestic, and leisure time-related domains and summing the MET-hours for all activities [21]. Dietary information was measured by frequency of intakes of fruits, vegetables and red meat. Cooking and heating fuel was categorized as non-cooking/heating, cleaner fuel, or solid/other fuel, respectively.

### Ascertainment of outcomes

The incidence information of the cohort participants was mainly obtained through passive follow-up methods, including the local health insurance system, disease surveillance registration system and chronic disease surveillance registration system, supplemented by active follow-up methods such as household investigations [17].

Incident COPD cases were coded by the International Classification of Disease 10th revision (ICD-10) as J41 to J44. Each incident is ascertained by medical records, original disease report cards, or official death certificates. The follow-up was completed on 31 December 2017.

### Statistical analysis

The continuous and categorical variables were presented as mean ± standard deviation and numerical (percentage), respectively. Unordered categorical variables and ordered categorical variables were compared by chi-square test and the rank sum test, respectively. The Fisher’s exact test was used for small examples and the Cochran-Armitage test was exploited for the trend examination of SES.

Participants contributed person-years in this analysis from enrollment into the baseline study to occurrence of the endpoint event of COPD, death, loss to follow-up or 31 December 2017, whichever occurred first. Cox proportional hazard regression models were used to estimate the association between SES and the risk of incidence of COPD by calculating hazard ratios (HR) and 95% confidence intervals (CI). Multivariate models were used to adjust covariates. Model 1 only adjusted for age and gender. Model 2 additionally included education, marital status, physical activity, BMI, alcohol consumption, frequency of red meat, frequency of fruits or vegetables, prevalent respiratory disease at baseline, and smoking status. Model 3 further adjusted passive smoking, cooking fuel pollution, and heating fuel pollution. Annual household income, education level and refrigerator use were treated as continuous variables to analyze the linear trend.

Several sensitivity analyses were conducted to examine the robustness of the association between household income and COPD in model 3. To avoid the potential for reverse causation, COPD cases were excluded from the initial three-year follow-up period. Individuals exhibiting airflow obstruction, as defined by FEV_1_/FVC < 0.7, were excluded as a further criterion for defining airflow obstruction. To avoid the potential confounding effect of prevalent asthma or diabetes on the development of COPD, participants with these conditions were excluded. Analysis of COPD hazard ratios (HRs) and 95% confidence intervals (CIs) for household income was further stratified by age, gender, physical activity, BMI, alcohol consumption, passive smoking, cooking fuel, and heating fuel. An interaction test was conducted by calculating the multiplicative interaction terms between household income and stratified variables.

R version 4.2.0 was used to perform the statistical analyses. Two-sided *p*-Values < 0.05 were considered to be statistically significant.

## Results

### Baseline characteristics

The characteristics of study participants at baseline were shown in Table 1. The age of all 45,484 participants was 51.67 ± 10.19 years, 57.9% were women. The proportion of participants with household income of < 10,000 yuan, 10,000–19,999 yuan, 20,000–34,999yuan, and ≥ 3,5000 yuan were 11.3%, 14.3%, 31.9%, and 42.6%, respectively. Compared with low household income, participants with high household income were more likely to be young, male, married, high BMI, high level of physical activity, current smokers, occasional or regular drinkers, consume red meat more frequently, eat fruits or vegetables daily, low prevalent respiratory disease at baseline, long term passive smoking, non-cooking and use cleaner fuel. For other SES indicators, participants who had high household income were more likely to have high education level, newly renovated flats within 5 years, private toilet, private phone, motor vehicle, holiday during last 5 years and long refrigerator use.

**Table 1:** Baseline characteristics of study participants by household income

Characteristics	Household income				*P* value
	< 10,000 yuan	10,000 ~ 19,999 yuan	20,000 ~ 34,999 yuan	≥ 35,000 yuan	
No of participants	5 124 (11.3)	6 485 (14.3)	14 502 (31.9)	19 373 (42.6)	
Age (years)	63.4 ± 8.4	53.5 ± 9.9	49.6 ± 9.3	49.5 ± 9.0	< 0.001
Female	3 359 (65.6)	3 929 (60.6)	8 729 (60.2)	10 326 (53.3)	< 0.001
Married	3 949 (77.1)	5 917 (91.2)	13 748 (94.8)	18 690 (96.5)	< 0.001
Education level					< 0.001
no formal school	3 369 (65.7)	2 322 (35.8)	3 766 (26.0)	3 775 (19.5)	
primary or middle school	1 711 (33.4)	3 880 (59.8)	9 537 (65.8)	12 552 (64.8)	
above high school	44 (0.9)	283 (4.4)	1 199 (8.2)	3 046 (15.7)	
Body mass index (kg/m^2^)	23.6 ± 3.3	23.9 ± 3.2	24.0 ± 3.2	24.4 ± 3.2	< 0.001
Physical activity (MET-h/day)	18.0 ± 14.1	26.1 ± 16.3	28.0 ± 15.2	26.1 ± 14.1	< 0.001
Alcohol consumption					< 0.001
non-drinker	3 690 (72.0)	4 166 (64.3)	8 865 (61.1)	9 805 (50.6)	
former drinker	215 (4.2)	162 (2.5)	257 (1.8)	289 (1.5)	
occasional drinker	594 (11.6)	1 112 (17.1)	2 958 (20.4)	5 331 (27.5)	
regular drinker	625 (12.2)	1 045 (16.1)	2 422 (16.7)	3 948 (20.4)	
Smoking^a^					< 0.001
never	3 556 (69.4)	4 100 (63.2)	9 127 (62.9)	10 844 (56.0)	
occasional	155 (3.0)	243 (3.7)	555 (3.8)	1 031 (5.3)	
ex-regular smoker	374 (7.3)	376 (5.8)	653 (4.5)	956 (4.9)	
current, < 10 cigarettes/d	677 (13.2)	1 124 (17.3)	2 742 (18.9)	4 232 (21.8)	
current, ≥ 10 cigarettes/d	223 (4.4)	391 (6.0)	879 (6.1)	1 387 (7.2)	
Frequency of red meat					< 0.001
rarely or never	886 (17.3)	673 (10.4)	949 (6.5)	847 (4.4)	
1 to 3 days/week	3 156 (61.6)	3 385 (52.2)	7 001 (48.3)	7 986 (41.2)	
≥ 4 days/week	1 082 (21.1)	2 427 (37.4)	6 552 (45.2)	10 540 (54.4)	
Prevalent respiratory disease at baseline^b^	77 (1.5)	95 (1.5)	157 (1.1)	267 (1.4)	0.027
Eating fruits or vegetables daily	5 087 (99.3)	6 445 (99.4)	14 429 (99.5)	19 295 (99.6)	0.015
Passive smoking					< 0.001
never lived with smoker	854 (16.7)	957 (14.8)	1 892 (13.0)	2 733 (14.1)	
lived with smoker for < 20 y	681 (13.3)	854 (13.2)	1 710 (11.8)	2 378 (12.3)	
lived with smoker for ≥ 20 y	3 589 (70.0)	4 674 (72.0)	10 900 (75.2)	14 262 (73.6)	
Cooking fuel^a^					< 0.001
non-cooking	595 (11.6)	1 056 (16.3)	2 638 (18.2)	4 851 (25.0)	
cleaner fuel	1 950 (38.1)	2 887 (44.5)	6 437 (44.4)	8 447 (43.6)	
solid/other fuel	1 475 (28.8)	882 (13.6)	1 326 (9.1)	898 (4.6)	
Heating fuel					< 0.001
non-heating	4 730 (92.3)	5 782 (89.2)	12 152 (83.8)	13 705 (70.7)	
cleaner fuel	393 (7.7)	702 (10.8)	2 339 (16.1)	5 652 (29.2)	
solid/other fuel	1 (0.0)	1 (0.0)	11 (0.1)	16 (0.1)	
Has newly renovated flats within 5 years	2 938 (57.3)	4 357 (67.2)	10 453 (72.1)	13 651 (70.5)	< 0.001
Has private toilet	3 340 (65.2)	5 680 (87.6)	13 937 (96.1)	19 121 (98.7)	< 0.001
Has private phone	2 677 (52.2)	5 873 (90.6)	14 349 (98.9)	19 327 (99.8)	< 0.001
Has motor vehicle	921 (18.0)	3 612 (55.7)	11 856 (81.8)	17 789 (91.8)	< 0.001
Had holiday during last 5 years	80 (1.6)	232 (3.6)	637 (4.4)	2 489 (12.8)	< 0.001
Refrigerator use (years)					< 0.001
0	3 439 (67.1)	2 482 (38.3)	2 936 (20.2)	1 170 (6.0)	
1~	1 057 (20.6)	2 306 (35.6)	6 347 (43.8)	6 849 (35.4)	
10~	562 (11.0)	1 379 (21.3)	4 039 (27.9)	8 179 (42.2)	
20~	66 (1.3)	318 (4.8)	1 180 (8.1)	3 175 (16.4)	

MET = metabolic equivalents of task

^a^Percentages in category do not add up to 100% because some participants did not answer

^b^Prevalent respiratory disease include pulmonary tuberculosis and asthma

### Association of SES with COPD

A total of 524 incident cases of COPD were identified during a median follow-up of 11.2 years. The results of the adjusted multivariate models were demonstrated in Tables 2 and 3. Household income was inversely associated with the risk of COPD (*P*_trend_<0.005) and higher COPD incidence was observed in participants with lower annual household income compared with those higher household income (4.300, 1.362 and 0.713 vs. 0.347 per 1000 person-years, Table 2). The adjusted hazard ratios (95% confidence intervals) for incident COPD were 0.88 (0.69–1.14), 0.77 (0.60–0.99), and 0.42 (0.31–0.57) for participants with annual household income of 10,000 ~ 19,999 yuan, 20,000 ~ 34,999 yuan and ≥ 35,000 yuan respectively, in comparison to participants with an annual household income < 10,000 yuan. We conducted a stratification analysis by smoking to investigate the effect of SES on COPD risk in the subgroups. After adjusting for potential confounders, consistent associations were observed between household income and COPD in smokers and non-smokers. However, compared with the smokers, a stronger strength of association between household income and COPD was observed in the non-smokers (Table 2).

**Table 2:** Adjusted hazard ratios (HRs) for COPD by household income

	Household income				*P* for trend
	< 10,000 yuan	10,000 ~ 19,999 yuan	20,000 ~ 34,999 yuan	≥ 35,000 yuan	
**Overall (** ***n*** ** = 45 484)**					
COPD incidence	238/55366.18	99/72664.35	115/161252.67	72/207211.82	
Model 1	1.00	0.78 (0.61–0.99)	0.64 (0.51–0.82)	0.34 (0.26–0.45)	< 0.001
Model 2	1.00	0.86 (0.67–1.10)	0.74 (0.58–0.95)	0.40 (0.30–0.54)	< 0.001
Model 3	1.00	0.88 (0.69–1.14)	0.77 (0.60–0.99)	0.42 (0.31–0.57)	< 0.001
**Smoker (** ***n*** ** = 17 857)**					
COPD incidence	110/16033.63	53/26018.23	56/58909.28	49/90156.85	
Model 1	1.00	0.86 (0.61–1.20)	0.71 (0.50-1.00)	0.50 (0.34–0.72)	< 0.001
Model 2	1.00	1.02 (0.72–1.45)	0.84 (0.59–1.21)	0.60 (0.41–0.89)	0.010
Model 3	1.00	1.02 (0.72–1.46)	0.85 (0.59–1.23)	0.61 (0.41–0.91)	0.015
**Never smoker (** ***n*** ** = 27 627)**					
COPD incidence	128/39332.54	46/46646.12	59/102343.39	23/117054.96	
Model 1	1.00	0.72 (0.51–1.02)	0.61 (0.44–0.86)	0.22 (0.14–0.35)	< 0.001
Model 2	1.00	0.74 (0.52–1.06)	0.69 (0.49–0.98)	0.26 (0.16–0.42)	< 0.001
Model 3	1.00	0.78 (0.55–1.11)	0.73 (0.51–1.03)	0.28 (0.17–0.46)	< 0.001

Multivariate models were adjusted for: model 1: age (years); sex (male or female); model 2: additionally included education (no formal school, primary or middle school, or above high school); marital status (married, widowed or divorced or separated, or never married); physical activity (MET(metabolic equivalent of task)h/day); body mass index (kg/m^2^); alcohol consumption (non-drinker, former drinker, occasional drinker, or regular drinker); frequency of red meat (≥ 4 days/week, 1 to 3 days/week, or rarely or never); frequency of fruits or vegetables (daily or no daily); prevalent respiratory disease at baseline (presence or absence); smoking (never, occasional, ex regular smoker, current < 10 cigarettes/d, or current ≥ 10/d); model 3: additionally included passive smoking (never lived with smoker, lived with smoker for < 20 y, or lived with smoker for ≥ 20 y); cooking fuel (non-cooking, cleaner fuel, or solid/other fuel); heating fuel (non-heating, cleaner fuel, or solid/other fuel)

**Table 3:** Adjusted hazard ratios (HRs) for COPD by other socioeconomic indicators

	COPD incidence	HR (95% CI)		
		Model 1	Model 2	Model 3
**Education level**				
no formal school	306/146098.28	1.00	1.00	1.00
primary or middle school	197/301751.13	0.58 (0.47–0.72)	0.59 (0.47–0.73)	0.61 (0.49–0.77)
above high school	21/48645.61	0.34 (0.21–0.53)	0.39 (0.24–0.61)	0.45 (0.28–0.73)
*P* for trend		< 0.001	< 0.001	< 0.001
**Has newly renovated flats within 5 years**				
No	176/147062.4	1.00	1.00	1.00
Yes	348/349432.6	1.28 (1.07–1.54)	1.23 (1.02–1.49)	1.28 (1.06–1.54)
**Has private toilet**				
No	137/36855.93	1.00	1.00	1.00
Yes	387/459639.08	0.61 (0.50–0.75)	0.71 (0.58–0.88)	0.77 (0.62–0.96)
**Has private phone**				
No	174/34905.18	1.00	1.00	1.00
Yes	350/461589.83	0.59 (0.49–0.72)	0.71 (0.57–0.87)	0.75 (0.61–0.93)
**Has motor vehicle**				
No	323/123768.7	1.00	1.00	1.00
Yes	201/372726.3	0.67 (0.55–0.82)	0.71 (0.58–0.87)	0.72 (0.59–0.87)
**Had holiday during last 5 years**				
No	499/459640.73	1.00	1.00	1.00
Yes	25/36854.29	0.63 (0.42–0.95)	0.87 (0.57–1.31)	0.95 (0.62–1.44)
**Refrigerator use (years)**				
0	289/111180.53	1.00	1.00	1.00
1~	138/182695.88	0.61 (0.49–0.75)	0.64 (0.52–0.80)	0.66 (0.53–0.83)
10~	70/153863.97	0.36 (0.28–0.47)	0.39 (0.30–0.51)	0.41 (0.31–0.54)
20~	27/48754.64	0.31 (0.21–0.46)	0.37 (0.24–0.58)	0.40 (0.26–0.62)
*P* for trend		< 0.001	< 0.001	< 0.001

Multivariate models were adjusted for: model 1: age (years); sex (male or female); model 2: additionally included education (no formal school, primary or middle school, or above high school); marital status (married, widowed or divorced or separated, or never married); physical activity (MET(metabolic equivalent of task)h/day); body mass index (kg/m^2^); alcohol consumption (non-drinker, former drinker, occasional drinker, or regular drinker); frequency of red meat (≥ 4 days/week, 1 to 3 days/week, or rarely or never); frequency of fruits or vegetables (daily or no daily); prevalent respiratory disease at baseline (presence or absence); smoking (never, occasional, ex regular smoker, current < 10 cigarettes/d, or current ≥ 10/d); model 3: additionally included passive smoking (never lived with smoker, lived with smoker for < 20 y, or lived with smoker for ≥ 20 y); cooking fuel (non-cooking, cleaner fuel, or solid/other fuel); heating fuel (non-heating, cleaner fuel, or solid/other fuel)

Absolute COPD incidence rates were 2.094, 0.653, and 0.437 per 1000 person-years for the categories ‘no formal school’, ‘primary or middle school’, and ‘above high school’, respectively.

Compared with participants who attained no formal school, the HRs (95% CIs) for COPD incidence were 0.61 (0.49–0.77) and 0.45 (0.28–0.73) for primary or middle school and above high school (*P* < 0.05 for trend). As the education level increased, the risk of COPD incidence decreased accordingly. Similarly, the HRs (95% CIs) for 1~, 10~,20 ~ years of using refrigerator were 0.66 (0.53–0.83), 0.41 (0.31–0.54), and 0.40 (0.26–0.62) respectively compared to those never used refrigerator, which indicated an inversely association between years of refrigerator use with the risk of COPD (*P* < 0.05 for trend). Moreover, compared to participants having no private toilet, private phone and motor vehicle, the HRs (95% CIs) for COPD incidence were 0.77 (0.62–0.96), 0.75 (0.61–0.93), and 0.72 (0.59–0.87) for those having these items respectively. Whereas, the adjusted HR (95% CI) of those having newly renovated flats within 5 years was 1.28 (1.06–1.54) for the incidence of COPD compared to participants who did not have these, which suggested a positive association between having newly renovated flats within 5 years and the risk of COPD incidence. Having holiday last 5 years was a protective factor for the risk of COPD incidence when adjusted age and gender in model 1 (HR = 0.63, 95%CI: 0.42–0.95). However, there was no statistically significant association observed between having holiday last 5 years and COPD risk after adjusting for potential confounders in model 2 and model 3.

### Stratified analyses

**Fig. 1:**
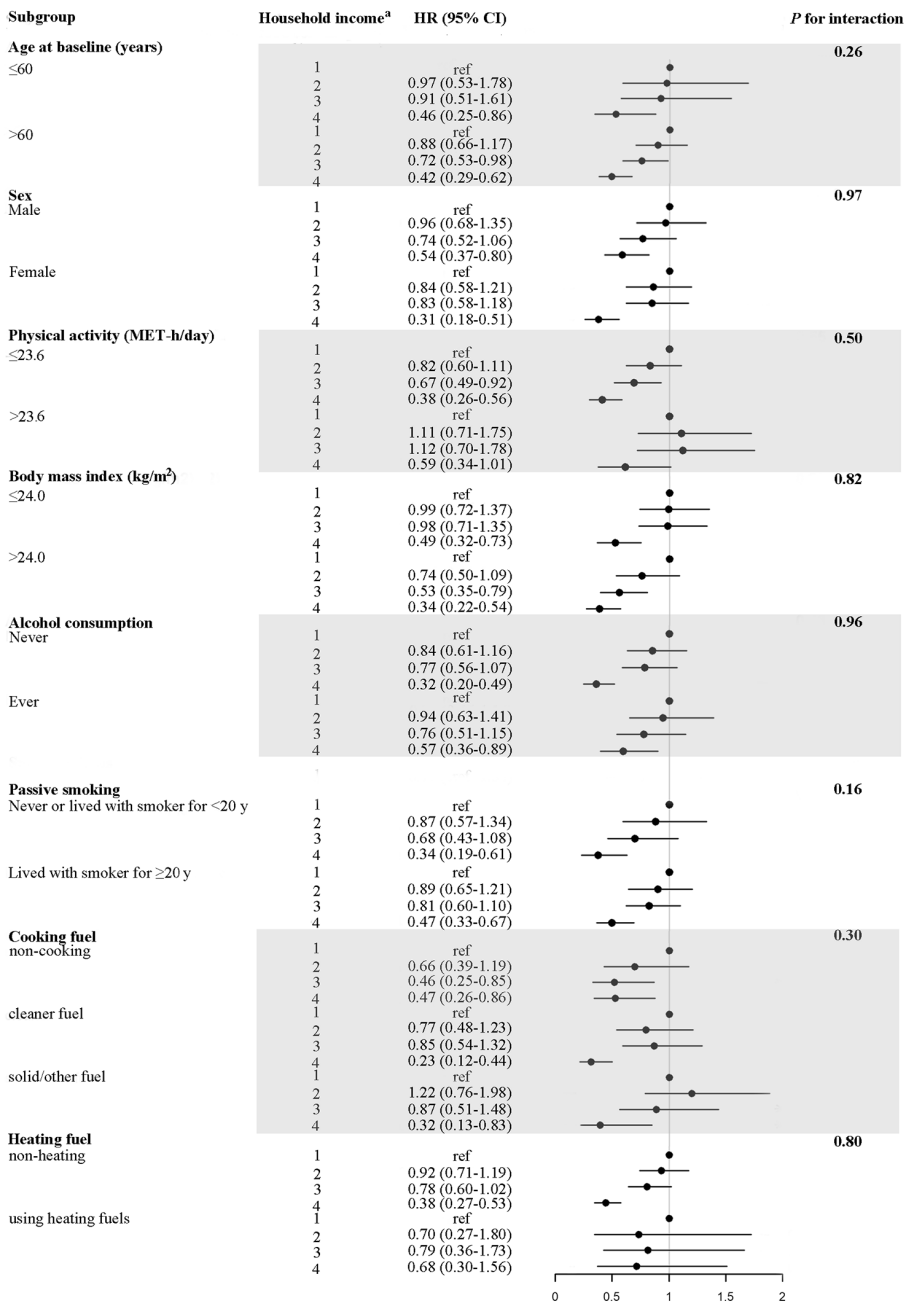
Stratified analysis of COPD hazard ratios (HRs) and 95% confidence intervals (CIs) for household income among all participants. ^a^Household income (< 10,000 yuan, 10,000–19,999 yuan, 20,000–34,999yuan, ≥ 35,000 yuan). Models were adjusted for age (years); sex (male or female); education (no formal school, primary or middle school, or above high school); marital status (married, widowed or divorced or separated, or never married); physical activity in MET(metabolic equivalent of task)-h/day; body mass index (kg/m^2^); alcohol consumption (non-drinker, former drinker, occasional drinker, or regular drinker); frequency of red meat (≥ 4 days/week, 1 to 3 days/week, or rarely or never); frequency of fruits or vegetables (daily or no daily); prevalent respiratory disease at baseline (presence or absence); smoking (never, occasional, ex regular smoker, current < 10 cigarettes/d, or current ≥ 10/d); passive smoking (never lived with a smoker, lived with a smoker for < 20 years, or lived with a smoker for ≥ 20 years); cooking fuel (non-cooking, cleaner fuel, or solid/other fuel); and heating fuel (non-heating, cleaner fuel, or solid/other fuel)

For the impact of household income on the COPD risk, a stratification analysis was conducted by age, sex, physical activity, BMI, alcohol consumption, passive smoking, cooking fuel and heating fuel. A consistent association was detected among household income and COPD risk in the subgroups as the interaction effects were not statistically significant (Fig. 1).

### Sensitivity analysis

Several sensitivity analyses were conducted by excluding COPD cases in the first 3 years of follow-up, excluding participants who had airflow obstruction with FEV_1_/FVC < 0.7, and excluding participants who had prevalent asthma or prevalent diabetes for the association between household income and COPD based on model 3. The estimated risk results did not change materially in the sensitivity analyses as all *P* trend < 0.05 (Table 4).

**Table 4:** Sensitivity analysis for the association between household income and COPD (HR and 95% CI)

	Household income				*P* for trend
	< 10,000 yuan	10,000 ~ 19,999 yuan	20,000 ~ 34,999 yuan	≥ 35,000 yuan	
Excluding cases in the first three years (*n* = 45 462)	1.00	0.85 (0.66–1.11)	0.77 (0.60–0.99)	0.42 (0.30–0.57)	< 0.001
Excluding FEV1/FVC < 0.7 definition for airflow obstruction (*n* = 45 260)	1.00	0.91 (0.71–1.18)	0.78 (0.60–1.01)	0.45 (0.33–0.61)	< 0.001
Excluding prevalent asthma (*n* = 45 323)	1.00	0.89 (0.69–1.14)	0.78 (0.61–1.01)	0.44 (0.32–0.60)	< 0.001
Excluding prevalent diabetes (*n* = 43 067)	1.00	0.91 (0.70–1.17)	0.79 (0.61–1.02)	0.44 (0.32–0.60)	< 0.001

Results were based on model 3

## Discussion

### Principal findings

We examined the associations of several SES indicators with the risk of COPD incidence in this large, prospective population-based cohort study in Suzhou, China. There was an inverse association between annual household income and the risk of COPD, which was stronger in the never smokers. Compared with annual household income < 10,000 yuan, household income ≥ 3,5000 yuan showed a 58%, 39%, and 72% reduction in the risk of COPD among the overall participants, smokers and never smokers, even after controlling the potential confounders. Similar associations were also found in other SES indicators (having high education level, private toilet, private phone, motor vehicle, and long refrigerator use). This might indicate that high SES is a protective factor for COPD.

### Comparison with other studies

Our findings on the association between SES and COPD in general adults are consistent with several previous studies [12, 16]. A prospective cohort study in Korea was performed and examined the contribution of socioeconomic disparity to all-cause mortality in COPD patients [16]. Similarly, a multiple population-based study also found that lower education, lower household income, and lower composite SES index were associated with COPD among low- and middle-income countries [22].

The potential mechanism may be that SES is related to the extent to which individuals are able to access health resources, including health knowledge, health behaviors, and healthcare services [23, 24]. Those with a low SES may engage in unhealthy behaviors, consume poor nutrition, and lack access to quality healthcare services, which can collectively increase the risk of developing COPD and result in poorer health outcomes. Furthermore, individuals with a low SES are more susceptible to environmental risk factors for COPD, including exposure to outdoor and indoor air pollution [25]. For example, residents with low SES may be exposed to environmental tobacco smoke, use non-clean fuel for cooking and heating, live in poorly ventilated dwellings, and be subjected to airborne pollutants due to occupational activities. In China, the dearth of hygienic domestic fuels and inadequate kitchen ventilation may contribute to indoor air pollution, which has been identified as a threat to women with COPD [26].

Although tobacco smoking was one of the main risk factors of COPD, recent researches recognized the importance of non-smoking-related risk factors for COPD [27]. Epidemiological studies show that about half of all COPD cases in the world are never smokers, which means that we should also consider other conditions such as air pollution, environmental tobacco smoke, infectious diseases, and low SES [28]. Meanwhile, COPD patients in never-smokers may have relatively mild chronic respiratory symptoms compared with those smokers [28]. In this study, the incidence of COPD in never smokers was lower than smokers, but the protect effect of household income for COPD was stronger in never smokers. The disparity of the smoking prevalence and frequency among different income groups reflects the inequalities in the initiation and cessation of smoking, and it deserves wider attention [29].

SES is defined as a broad concept to reflect social and economic status, which is often measured by income, education and occupation [30]. To identify and capture more potentially socioeconomic characteristics, this study included multiple SES measures such as private toilet, private phone, motor vehicle, refrigerator use, holiday, and new renovated flats. It seems that to some extent, income, education and private consumption level can represent both consumption power and economic strength. Meanwhile, having newly renovated flats within 5 years was observed to be related to a 28% increased risk of COPD incident, perhaps due to the indoor pollutants from new renovation [31]. In this study, having holiday during last 5 years was irrelevant with the risk of COPD, which indicated that the indicator of whether there is a holiday can not reflect well on SES, as people with lower SES may also have holiday.

### Public health impact

The observed SES disparity in COPD indicates a pressing need for further research on developing and implementing strategies to improve the health of individuals with low SES, with a particular focus on China. Over the past decades, the rapid economic development in China has contributed to an exacerbation of the gap between the rich and the poor, which has in turn exacerbated health inequalities [32, 33]. It is imperative to gain a deeper comprehension of the ways in which social and economic factors, both directly and indirectly, influence health disparities and the outcomes of COPD in different demographic groups. Additionally, it is crucial to elucidate the relationship between various SES measures and the occurrence and developmental outcomes of COPD. It is therefore recommended that appropriate strategies be implemented to reduce the burden of disease and social inequality caused by SES differences in COPD outcomes.

### Strengths and limitations

The major strengths of this study are the well-established population-based prospective cohort study with a large sample size of population. We controlled the potential confounding including main risk factors of COPD such as smoking and indoor air pollution in the analyses and performed the stratified analyses with sufficient statistical power. In addition to the most commonly used indicators of SES such as household income and education, we also included several other indicators such as private phone, toilet, motor vehicle and refrigerator use, which had not been seen in other research analysis. Moreover, a series of sensitivity analyses were also conducted to examine the robustness of the findings.

Meanwhile, there were several limitations should be concerned. First, data on the SES indicators in this study were mainly self-reported, which may increase the probability of misclassification bias. Second, the SES indicators were only measured once at baseline, so the SES trajectories during the follow-up could not be captured. However, the re-survey implemented after the baseline showed the consistency between the baseline and re-survey for SES [17]. Third, although we conducted multivariate, stratified and sensitivity analyses to adjust risk factors for COPD, possible residual confounding factors may not be completely eliminated. In addition, detailed information on the amount of cigarettes smoked (e.g., number of pack-years) was not available to allow for more detailed adjustments for smoking factors in the model. Additionally, COPD incidents in the follow-up were mainly identified by monitoring systems including chronic disease, death registries and health insurance system, thus some COPD cases might still be undetected. It should be noted that the subjects of this cohort are drawn from the general population of the community, but the study area is limited to Suzhou City. It would be prudent to exercise caution when extrapolating the findings to the broader Chinese population.

## Conclusion

This prospective cohort study in Chinese adults provided evidence that SES, including household income, education, private toilet, private motor vehicle, private phone and refrigerator use, is adversely associated with the incidence of COPD. People with poor social and economic conditions have more health risks with COPD. Further studies were needed to explore the main determinants of excess risk for socioeconomically disadvantaged individuals with or at risk for COPD. Investigations and development of COPD prevention strategies and government policies for individuals with disadvantaged SES to reduce the socioeconomic inequity in COPD have important public health implications.

## Data Availability

The datasets used and/or analyzed during the current study are available from the corresponding author on reasonable request.

## Abbreviations

SES: socioeconomic status
COPD: chronic obstructive pulmonary disease
HR: hazard ratio
CI: confidence intervals
CKB: China Kadoorie Biobank
FEV1: forced expiratory volume at 1 s
FVC: forced vital capacity
LLN: lower limit of normal
MET: metabolic equivalent task
BMI: body mass index
